# Investigating the trend of demographic changes, mortality, clinical and paraclinical findings of patients hospitalized in the Corona ward, before and after the start of general vaccination of COVID-19

**DOI:** 10.1186/s12879-024-09279-z

**Published:** 2024-05-13

**Authors:** Reza Morovatshoar, Kiavash Hushmandi, Sara Orouei, Seyed Hassan Saadat, Rasoul Raesi

**Affiliations:** 1 Molecular Medicine Research Center, Hormozgan Health Institute, Hormozgan University of Medical Sciences, Bandar Abbas, Iran; 2 Nephrology and Urology Research Center, Clinical Sciences Institute, Baqiyatallah University of Medical Sciences, Tehran, Iran; 3 Department of psychology, North Tehran branch, Islamic Azad University, Tehran, Iran; 4 Department of Health Services Management, Mashhad University of Medical Sciences, Mashhad, Iran; 5 Department of Nursing, Torbat Jam Faculty of Medical Sciences, Torbat Jam, Iran

**Keywords:** Mortality, Morbidity, Vaccine, COVID-19, Hospital, General vaccination, Clinical, Findings, Demography

## Abstract

**Background:**

Prioritizing prevention over treatment has been a longstanding principle in the world health system. This study aims to compare the demographic changes, mortality, clinical, and paraclinical findings of patients hospitalized in the Corona ward before and after the start of general vaccination.

**Methods:**

This cross-sectional study utilized the simple random sampling method in 2022, analyzing 300 medical records of patients admitted to the Corona ward at 22 Bahman Khaf Hospital. Data were collected using a checklist with the help of the Medical Care Monitoring System and analyzed using SPSS-22 statistical software and Chi-square statistical test at a significance level of *p* < 0.05.

**Results:**

Before the start of general vaccination for COVID-19, the majority of patients were hospitalized in the Corona Intensive Care Unit (59.3%), aged between 51 and 65 years (47.3%), hospitalized for more than 3 days (54%), required intubation (49.3%), had SPO2 < 93% (60.7%), and exhibited common symptoms such as cough, shortness of breath, and loss of consciousness. Paraclinical findings included positive CRP, decreased lymphocytes, and ground glass opacity (GGO). After the start of general vaccination for COVID-19, most patients were hospitalized in the general care department of Corona (68%), aged between 36 and 50 years (47.3%), hospitalized for less than three days (66%), required intubation (20%), had SPO2 ≥ 93% (77.3%), and exhibited common symptoms such as weakness, headache, and body pain. Paraclinical findings were within the normal range.

**Conclusions:**

General vaccination for COVID-19 has significantly reduced patient mortality and morbidity. Health policymakers should prioritize general vaccination to achieve herd immunity and improve public health.

**Supplementary Information:**

The online version contains supplementary material available at 10.1186/s12879-024-09279-z.

## Background

Prioritizing prevention over treatment has been in the world health system for a long time. The sudden onset of the COVID-19 crisis reaffirmed this priority [1]. The consequences and costs caused by this disease had such a profound effect on the economy of countries in the world that it doubled the need for a vaccine as soon as possible. This issue clarified the importance of vaccine production technology as one of the strategic technological capabilities in the current century [2–4]. 

Governments have made many efforts to control and suppress this disease, including public education, social distancing policies, screening tests, and vaccine efforts [1, 5]. One of the most important global strategies to control and contain the epidemic of Kovid-19 was the vaccine. Vaccination is the most effective way to control infectious diseases, especially in high-risk groups. In addition to ensuring health and reducing damage and loss of life and ensuring public health, vaccination also leads to a reduction in medical expenses and pharmaceutical costs and as a result economic savings [6]. 

During the outbreak of COVID-19, after the clinical trial of a number of vaccines was confirmed, some people were skeptical about getting the vaccine and participating in the nationwide vaccination process. However, after the passage of time and the positive effects of the COVID-19 vaccine on reducing hospitalization and death due to COVID-19, many people showed a positive view and acceptance towards the vaccines approved by the World Health Organization [7–9]. 

The results of another study show that injecting two doses of the vaccine significantly reduces the risk of hospitalization. The role of the vaccine in increasing the body’s immune system is so impressive that other vaccines are also able to create immunity levels against COVID-19. In this regard, in a study, Wilcox et al. investigated the role of the flu vaccine in reducing the hospitalization of people during the outbreak of COVID-19. This study was conducted as a retrospective cohort on 6921 people. Surveys showed that 38% of these people had received the flu vaccine in 2019. The hospitalization rate of people who received the vaccine was very low, to the point that the probability of death of these people was also reduced by 24% [10]. 

The results of the studies indicate that the incidence of COVID-19 and the severity of its complications are related to the demographic characteristics of people. So that age is one of the risk factors related to contracting COVID-19. Compared to the young age group (20 to 29 years old), children and the elderly are more likely to be infected with COVID-19. Especially if children are in contact with a patient aged 30 to 39 years or 50 to 59 years, the chance of their infection increases [9, 11]. 

The death rate of the disease in February 2020 has been reported from 0.7% to more than 3% depending on the ability of health care [12]. However, in March 2020, the World Health Organization announced the death rate of this disease at 3.4% [13]. Although some studies have mentioned the fatality rate of the disease up to 5% [14]. The death rate varies by age. It ranges from 0.2% for ages 10 to 39, to 14.8% for ages over 80. It is 2.8% in men and 1.7% in women. Mortality in healthy people is 0.9% [15]. In Iran, according to a study, the overall mortality rate is 10.5% [16]. The average hospitalization in ICU is 12 days and the mortality rate is 24% [17]. 

Nationwide vaccination in Iran started on February 9, 2021 with the priority of medical personnel [18] and after that vaccination was done for high-risk groups such as the elderly and people with underlying diseases. Since the COVID-19 vaccination can be one of the most basic methods of preventing COVID-19 and also reducing mortality. And so far, no study has investigated the effect of COVID-19 vaccination on demographic changes, mortality, clinical and paraclinical findings of patients hospitalized in the corona ward before and after the start of vaccination. This research was carried out as an innovation with the aim of comparing the trend of demographic changes, mortality, clinical and paraclinical findings of patients hospitalized in the corona ward, before and after the start of general vaccination of COVID-19.

## Methods

### Study design and setting

This descriptive-analytical cross-sectional study was conducted in 2022 using the simple random sampling method by examining the medical records of 300 patients admitted to the Corona Ward at 22 Bahman Hospital in Khaf, located in the northeastern part of Iran.

### Study participants and sampling

Since the total number of hospitalized patients diagnosed with coronavirus was known in the investigated period, the sample size was 300 people using Cochran’s sample size formula with an error level of 5%. Finally, according to the inclusion and exclusion criteria of the study, by simple random sampling, the 150 medical records of patients with COVID-19 before the start of general vaccination and also the 150 medical records of patients with COVID-19 after the start of general vaccination were examined. 150 medical records were examined in patients with COVID-19 for the period after the start of general vaccination; all had a history of COVID-19 vaccination.

### Data collection tool and technique

At this stage, the researcher divided the data collection into two 6-month intervals according to the objectives of the study. The first period is related to before the start of general vaccination of COVID-19 (from May 21, 2020 to January 19, 2021). The second period is related to after the start of general vaccination of COVID-19 (from March 21, 2021 to November 21, 2021). A positive PCR test was considered as the criteria for entering the study, and the incompleteness of the file in terms of the information required for the research was considered as the exclusion criterion. Data collection was done using the Medical Care Monitoring System (MCMC) in the checklist. This checklist consists of three parts, which are: 1- Demographic characteristics (age, gender, Job, education level, marital status, place of residence, underlying diseases, history of smoking, inpatient department, length of hospitalization), 2- Clinical findings (intubation, saturation of peripheral oxygen (SpO2), treatment outcome, fever, cough, dyspnea, weakness, loss of consciousness (LOC), headache, body pain) and 3- paraclinical findings (positive CRP, lymphocytopenia, positive CRP and lymphocytopenia, ground-glass opacity (GGO), patchy consolidation).

Data were analyzed using SPSS version 22 statistical software. To compare the frequency distribution of the variables in the two groups of people under investigation before and after the start of general vaccination of COVID-19, the chi-square test was used. A significance level of *p* < 0.05 was considered.

## Results

In this research, 150 patients admitted to the corona ward before the start of general vaccination of COVID-19 and 150 patients admitted to the corona ward after the start of general vaccination of COVID-19 were examined. 150 medical records were examined in patients with COVID-19 for the period after the start of general vaccination; all had a history of COVID-19 vaccination. The comparison of the demographic characteristics of the examined patients before and after the start of general vaccination of COVID-19 showed that there was no significant difference in gender, occupation, education level, marital status and place of residence of the patients before and after the start of general vaccination of COVID-19 (*p* > 0.05). There was a significant difference in the age of patients before and after the start of general vaccination of COVID-19 (*p* < 0.001). So that most of the patients were 51–65 years old before the start of general vaccination of COVID-19, but after the start of general vaccination of COVID-19, most of the people were in the age group of 36–50. Most of the patients (59.3%) were hospitalized in the special ward before the general vaccination of COVID-19 and after the general vaccination of COVID-19 (68%) were hospitalized in the regular ward (*p* < 0.001). Also, before the start of general vaccination of COVID-19, the majority of patients (54%) were hospitalized for more than 3 days, and after the start of general vaccination of COVID-19, most of the patients (66%) were hospitalized for 3 days or less (*p* < 0.001) (Table 1).

**Table 1:** Comparison of the demographic characteristics of the investigated patients, before and after the start of general vaccination of COVID-19

Variable		Before vaccination	After vaccination	Statistical test result
		Number (percent)	Number (percent)	
Age	≤20 years	10 (6.7)	7 (4.7)	χ^2^ = 25.24 p < 0.001
	21–35 years	10 (6.7)	18 (12)	
	36–50 years	27 (18)	58 (38.7)	
	51–65 years	71 (47.3)	55 (36.7)	
	66–80 years	32 (21.3)	12 (8)	
Gender	Male	93 (62)	102 (68)	χ^2^ = 1.19 p = 0.28
	Female	57 (38)	48 (32)	
Job	Housekeeper	43 (28.7)	45 (30)	χ^2^ = 7.17 p = 0.13
	Employee	35 (23.3)	35 (23.3)	
	Self-employment	27 (18)	41 (27.3)	
	Retired	17 (11.3)	8 (5.3)	
	Unemployed	28 (18.7)	21 (14)	
Education level	Illiterate	56 (37.3)	45 (30)	χ^2^ = 1.81 p = 0.41
	Non-academic	55 (36.7)	61 (40.7)	
	Academic	39 (26)	44 (29.3)	
Marital status	Single	51 (34)	55 (36.7)	χ^2^ = 0.32 p = 0.57
	Married	99 (66)	95 (63.3)	
Place of residence	City	89 (59.3)	102 (68)	χ^2^ = 2.44 p = 0.12
	Village	61 (40.7)	48 (32)	
Inpatient department	General	61 (40.7)	102 (68)	χ^2^ = 22.58 p < 0.001
	Intensive	89 (59.3)	48 (32)	
length of hospitalization	≤3 days	69 (46)	99 (66)	χ^2^ = 12.18 p < 0.001
	>3 days	81 (54)	51 (34)	

Relative frequency of underlying diseases (diabetes, hypertension, kidney) and history of smoking in the examined patients, before and after the start of general vaccination for COVID-19, did not differ significantly (*p* > 0.05). 74 (49.3%) of the patients had intubation before the start of the general vaccination for COVID-19 and 30 (20%) of them had intubation after the start of the general vaccination for COVID-19 (*p* < 0.001). The Saturation of Peripheral Oxygen (SpO2) level of most of the patients was less than 93 before the general vaccination for COVID-19 (60.7%) and 93 or more after the general vaccination for COVID-19 (77.3%) (*p* < 0.001). The rate of the examined patients was 30.7% before the start of general vaccination for COVID-19, which decreased to 17.3% after the start of general vaccination for COVID-19 (*p* = 0.007). A comparison of clinical findings shows that there was no significant difference in fever in patients before and after COVID-19 general vaccination (*p* = 0.17). However, cough, dyspnea, and loss of consciousness (LOC) before the start of general vaccination for COVID-19 compared to after, and weakness, headache, and body pain after the start of general vaccination for COVID-19 were significantly more than before (*p* < 0.001) (Table 2).

**Table 2:** Comparison of the relative frequency of underlying diseases, history of smoking, intubation status, Saturation of Peripheral Oxygen (SpO2) level, treatment outcome, and clinical findings in the investigated patients before and after the start of general vaccination against COVID-19

Variable		Before vaccination	After vaccination	Statistical test result
		Number (percent)	Number (percent)	
Diabetes disease	No	65 (43.3)	69 (46)	χ^2^ = 0.22 p = 0.64
	Yes	85 (56.7)	81 (54)	
Hypertension disease	No	76 (50.7)	64 (42.7)	χ^2^ = 1.93 p = 0.17
	Yes	74 (49.3)	86 (57.3)	
kidney disease	No	147 (98)	149 (99.3)	χ^2^ = 1.01 p = 0.31
	Yes	3 (2)	1 (0.7)	
Smoking history	No	81 (54)	93 (62)	χ^2^ = 1.97 p = 0.16
	Yes	69 (46)	57 (38)	
Intubation	No	76 (50.7)	120 (80)	χ^2^ = 28.49 p < 0.001
	Yes	74 (49.3)	30 (20)	
Saturation of Peripheral Oxygen (SpO2)	<93	91 (60.7)	34 (22.7)	χ^2^ = 44.56 p < 0.001
	≥93	59 (39.3)	116 (77.3)	
Treatment outcome	Improvement and discharge	104 (69.3)	124 (82.7)	χ^2^ = 7.31 p = 0.007
	Death	46 (30.7)	26 (17.3)	
Fever	No	11 (7.3)	18 (12)	χ^2^ = 1.87 p = 0.17
	Yes	139 (92.7)	132 (88)	
Cough	No	0 (0)	54 (36)	χ^2^ = 65.85 p < 0.001
	Yes	150 (100)	96 (64)	
Dyspnea	No	7 (4.7)	123 (82)	χ^2^ = 182.66 p < 0.001
	Yes	143 (95.3)	27 (18)	
Weakness	No	149 (99.3)	127 (84.7)	χ^2^ = 21.92 p < 0.001
	Yes	1 (0.7)	23 (15.3)	
Loss of consciousness (LOC)	No	125 (83.3)	140 (93.3)	χ^2^ = 7.28 p = 0.007
	Yes	25 (16.7)	10 (6.7)	
Headache	No	139 (92.7)	117 (78)	χ^2^ = 12.89 p < 0.001
	Yes	11 (7.3)	33 (22)	
Body pain	No	150 (100)	120 (80)	χ^2^ = 33.33 p < 0.001
	Yes	0 (0)	30 (20)	

The comparison of laboratory and radiological findings in Table 3 shows that the laboratory findings of most patients before the start of general vaccination were positive CRP and lymphocytopenia and normal after the start of vaccination (*p* < 0.001). The findings of radiology were also normal in most patients before the start of general vaccination of COVID-19, Ground-glass opacity (GGO), and after the start of general vaccination (*p* < 0.001).

**Table 3:** Comparison of laboratory and radiological findings in the examined patients, before and after the start of general vaccination of COVID-19

Variable		Before vaccination	After vaccination	Statistical test result
		Number (percent)	Number (percent)	
Laboratory findings	Normal	49 (32.6)	102 (68)	χ^2^ = 39.25 p < 0.001
	positive CRP	10 (6.7)	2 (1.3)	
	Lymphocytopenia	12 (8)	4 (2.7)	
	positive CRP and Lymphocytopenia	79 (52.7)	42 (28)	
Radiological findings	Normal	50 (33.3)	118 (78.6)	χ^2^ = 62.59 p < 0.001
	Ground-glass opacity (GGO)	80 (53.3)	25 (16.7)	
	Patchy consolidation	20 (13.4)	7 (4.7)	

## Discussion

The present study was conducted with the aim of comparing the trend of demographic changes, mortality, clinical and paraclinical findings of patients admitted to the corona ward, before and after the start of general vaccination of COVID-19. The most important findings of the present study indicate that before the start of general vaccination for COVID-19, the majority of patients were hospitalized in the Corona Intensive Care Unit (59.3%), aged between 51 and 65 years (47.3%), hospitalized for more than 3 days (54%), required intubation (49.3%), had SPO2 < 93% (60.7%), and exhibited common symptoms such as cough, shortness of breath, and loss of consciousness. Paraclinical findings included positive CRP, decreased lymphocytes, and ground glass opacity (GGO). After the start of general vaccination for COVID-19, most patients were hospitalized in the general care department of Corona (68%), aged between 36 and 50 years (47.3%), hospitalized for less than three days (66%), required intubation (20%), had SPO2 ≥ 93% (77.3%), and exhibited common symptoms such as weakness, headache, and body pain. Paraclinical findings were within the normal range.

The findings of the present study showed that before the general vaccination of COVID-19, most of the patients hospitalized in corona wards were in the age range of 51–65 years. However, after the start of general vaccination against COVID-19, most of the patients hospitalized in corona wards were in the age range of 36–50 years.

In line with this finding of the present study, the results of the studies A. Christie et al. (2021) [19], M. L. Salomão et al. (2022) [20] as well as Emre Özgen et al. (2023) [21] showed that general vaccination of COVID-19 was associated with a change in the age range of patients hospitalized in corona wards. So that after the start of general vaccination of COVID-19, most of the patients hospitalized in corona wards were in the age group of less than 50 years.

But the results of the study K. Dooling et al. (2021) [22] as well as the results of the study by H. Rossman et al. (2021) [23] are not in line with this finding of the present study. So that the results of their studies showed that the general vaccination of COVID-19 is not related to the change in the age range of hospitalized patients. Also, most of the patients admitted to the Corona wards were elderly people over 60 years old. This discrepancy in research findings can be related to the different geographical environment in the studies as well as the type and number of hospitalized patients.

The findings of the present study showed that most of the patients were hospitalized in the Corona Intensive Care Department before the general vaccination of COVID-19. After the start of the general vaccination of COVID-19, most of the patients were hospitalized in the normal care department of Corona. The results of the study of M. Moffa et al. (2022) [24] as well as the results of the study M. Fogolari et al. (2022) [25] is in line with this finding of the present study. The results of their studies showed that after the start of general vaccination of COVID-19, most of the patients were hospitalized in the general care departments for corona patients. So that the number of patients hospitalized in the special corona wards has decreased.

But the results of the study of M. Özsoy et al. (2023) [26] as well as the results of the study of B. Ngo et al. (2021) [27] is not in line with this finding of the present study. So that the results of their study showed that there is no connection between the start of general vaccination of COVID-19 and the type of inpatient department of corona patients. This discrepancy in research findings can be related to the different geographical environment in the studies as well as the type and number of hospitalized patients.

The findings of the present study showed that before the general vaccination of COVID-19, most of the patients were hospitalized for more than 3 days in the care units for corona patients. Also, the findings showed that after the start of general vaccination for COVID-19, most of the patients were hospitalized in the care units for corona patients for less than three days.

In line with this finding of the current research, the results of the study of M. Tenforde et al. (2021) [28] and the results of the study by Aakashneel Bhattacharya et al. (2021) [29] showed that the start of general vaccination of COVID-19 is related to the number of days of hospitalization of patients in corona wards. so that after the start of the general vaccination of COVID-19, the number of days of hospitalization of patients in corona wards has decreased.

However, the results of the study of G. Suleyman et al. (2022) [30] and the results of the study of Anshuman Srivastava et al. (2022) [31] are not in line with this finding of the present study. So that the results of their study showed that there is no relationship between the start of general vaccination for COVID-19 and the number of days of hospitalization of patients in the care departments for corona patients.

The findings of the present study showed that the number of patients requiring intubation had decreased after the start of general vaccination against COVID-19. In line with this finding of the present study, the results of the study by C. Bezzio et al. (2020) [32] as well as the results of the study by Cristiane de Freitas Paganoti et al. (2022) [33] showed that the start of general vaccination of COVID-19 has been associated with a decrease in the need for intubation and also a decrease in the need for hospitalization in special corona wards.

The findings of the present study showed that the percentage of oxygen saturation (SPO2) in most patients was less than 93% before the start of the general vaccination of COVID-19 and more than 93% after the start of the general vaccination of COVID-19. In line with this finding of the present study, the results of Linzy Houchen-Wolloff et al. (2021) [34] as well as the results of the study of Ulfa Husnul Fata et al. (2022) [35] showed that the start of general vaccination of COVID-19 was associated with an increase in the percentage of oxygen saturation in patients hospitalized in corona wards.

The findings of the present study showed that the mortality rate in patients hospitalized in the care units for Corona patients decreased after the start of general vaccination for COVID-19. The results of the study by R. Kempker et al. (2022) [36] and the results of the study by P. Moreno-Nunez et al. (2022) [37] are in line with this finding of the present study. The results of their study showed that the start of general vaccination for COVID-19 was associated with a decrease in mortality in patients hospitalized in COVID-19 wards.

The findings of the present study showed that before the general vaccination of COVID-19, cough, dyspnea, and loss of consciousness were among the common symptoms of patients hospitalized in corona wards. However, after the start of the general vaccination of COVID-19, general weakness, headache, and body pain were among the common symptoms of patients hospitalized in corona wards. The results of the study of Zunaira Khan et al. (2022) [38] are in line with this finding of the present study, as the results of their study showed that: after the start of the general vaccination of COVID-19, general weakness, headache, and acute myelopathy were among the common symptoms of patients hospitalized in corona wards.

Also, the results of the study by L. Bonifácio et al. (2022) [39] are in line with this finding of the present study. The results of their study showed that: before the general vaccination of COVID-19, common symptoms like cough, dyspnea, and loss of consciousness were common, but after the vaccination, general weakness, headache, and muscle weakness emerged.

The findings of the present study showed that the laboratory results of most of the patients hospitalized in the care units for corona patients before the start of general vaccination of COVID-19 included positive CRP and a decrease in lymphocytes. However, after the start of the general vaccination of COVID-19, the laboratory findings of most of the patients hospitalized in the care units for corona patients were normal.

The results of the study of H. Fu et al. (2020) [40] are in line with this finding of the present study, in such a way that the results of their study showed that: after the start of general vaccination, CRP levels decreased significantly and lymphocyte counts increased in COVID-19 patients. Also, the results of the study of H. Akbari et al. (2020) [41] are in line with this finding of the present study. The results of their study showed that: before the start of general vaccination, most COVID-19 patients had a decrease in lymphocytes and an increase in CRP, but after the start of vaccination, these results reversed.

The findings of the present study showed that the radiology results of most of the patients hospitalized in the care departments of Corona patients before the start of general vaccination of COVID-19 were ground glass opacity (GGO). However, after the start of general vaccination against COVID-19, the radiology results of most of the patients hospitalized in the care units for corona patients were normal.

The results of the study of Mamatha Reddy D. Cozzi et al. (2021) [42] are in line with this finding of the present study in such a way that the results of their study showed that: before the start of general vaccination against COVID-19, most hospitalized patients had ground-glass opacities (GGO) on CT, but after the vaccination, GGO results decreased. Also, the results of the study of Jufriadif Na’am et al. [43] (2021) are in line with this finding of the present study in such a way that the results of their study showed that: before the start of general vaccination against COVID-19, most hospitalized patients had radiology results showing ground glass opacity (GGO) in their thorax.

### Limitations and recommendation

Among the limitations of the study were the short study period and the small number of samples under investigation. It is suggested to conduct future studies over a longer period of time and with a larger sample size. Another limitation of the current study was the newness of the MCMC system, which was associated with limitations such as not recording a number of variables. Therefore, access to all demographic, clinical, and paraclinical variables of the patients was not possible.

## Conclusion

The research findings reveal significant shifts in the clinical profile of COVID-19 patients before and after the general vaccination for COVID-19. These findings have several implications for health policymakers. First, they underscore the importance of prioritizing COVID-19 vaccination, particularly for high-risk groups such as the elderly and those with comorbidities. Second, the shift in patient demographics and clinical presentation highlights the need for ongoing public awareness campaigns to promote vaccination uptake and address concerns about vaccine safety and efficacy.

Third, the research emphasizes the critical role of healthcare providers in initiating discussions about COVID-19 vaccines with patients and acting as role models by getting vaccinated themselves.

In light of these findings, health policymakers should continue to prioritize and promote COVID-19 vaccination efforts, provide training and guidelines for healthcare providers, and monitor vaccine effectiveness and safety. Additionally, efforts should be made to address disparities in vaccination uptake and ensure that accurate information about COVID-19 vaccines is accessible to all segments of the population. By implementing these recommendations, policymakers can contribute to the ongoing efforts to mitigate the impact of the COVID-19 pandemic and protect public health.

### Electronic supplementary material

Below is the link to the electronic supplementary material.

Supplementary Material 1

Supplementary Material 2

Supplementary Material 3

Supplementary Material 4

## Data Availability

The data that support the findings of this study are available from the corresponding author upon reasonable request.
