# Supplementary material for: Investigating the trend of demographic changes, mortality, clinical and paraclinical findings of patients hospitalized in the Corona ward, before and after the start of general vaccination of COVID-19
Source: BMC Infect Dis. 2024 May 13;24:488. doi: 10.1186/s12879-024-09279-z (PMC11089665; doi:10.1186/s12879-024-09279-z)
Supplement: Supplementary file 1 — Supplementary Material 1 [file 12879_2024_9279_MOESM1_ESM.docx]

Investigating the trend of demographic changes, mortality, clinical and paraclinical findings of patients hospitalized in the Corona ward, before and after the start of general vaccination of COVID-19 (a retrospective study)

Running title: Investigation of hospitalized patients in the COVID-19 ward, before and after the general vaccination

Reza Morovatshoar^1^, Kiavash Hushmandi^2^, Sara Orouei^3^, Seyed Hassan Saadat^2^, Rasoul Raesi^4,5*^

1- Molecular Medicine Research Center, Hormozgan Health Institute, Hormozgan University of Medical Sciences, Bandar Abbas, Iran. Email: [Rezamorovatshoar1996@gmail.com](mailto:Rezamorovatshoar1996@gmail.com). ORCID: 0000-0002-0247-7924

2- Nephrology and Urology Research Center, Clinical Sciences Institute, Baqiyatallah University of Medical Sciences, Tehran, Iran. Email: [Kiavash.hushmandi@gmail.com](mailto:Kiavash.hushmandi@gmail.com). Email: [saadat350@gmail.com](mailto:saadat350@gmail.com)

3- Department of psychology, North Tehran branch, Islamic Azad University, Tehran, Iran. Email: [sara_oruei@yahoo.com](mailto:sara_oruei@yahoo.com)

4- Department of Health Services Management, Mashhad University of Medical Sciences, Mashhad, Iran. Email: [Raesi.br881@gmail.com](mailto:Raesi.br881@gmail.com)

5- Department of Nursing, Torbat Jam Faculty of Medical Sciences, Torbat Jam, Iran.

**^*^ Corresponding author: Rasoul Raesi, Email:** [Raesi**.**br881@gmail**.**com](mailto:Raesi.br881@gmail.com)**. ORCID:** 0000-0000-5998-5209

**Declarations**

**Ethics approval and consent to participate**

In terms of ethical considerations, the present study was ethical in two respects: first, all the participants volunteered to participate in the study, and secondly, keeping in mind the principles of confidentiality and secrecy, participants were assured that all information would remain confidential and that the results would be reported in a general manner. All procedures performed in the study involving human participants were by the ethical standards of the institutional and national research committee and with the 1975 Helsinki Declaration and its later amendments or comparable ethical standards. This study was approved by the Biomedical Research Ethics Committee of Mashhad University of Medical Sciences, which issued the study’s code of ethics (IR.MUMS.REC.1401.050.).

**Consent for publication**

In order to comply with ethical considerations in this research, the information of the participants was kept confidential and other people were not able to access this information. The names and surnames of the participants were not used for data collection, and data collection was done after obtaining the code of ethics from Mashhad University of Medical Sciences.

**Availability of data and materials**

The data that support the findings of this study are available from the corresponding author upon reasonable request.

**Competing interests**

The authors declare no conflict of interest, financial or otherwise.

**Funding**

This research was done with the financial support of Mashhad University of Medical Sciences.

**Authors' contributions**

"RM and KH analyzed and interpreted the data. RR, SO, SHS contributor in writing the manuscript. All authors read and approved the final manuscript."

**Acknowledgements**

We would like to express our sincere thanks and appreciation to the honorable research assistant of Mashhad University of Medical Sciences, the honorable officials of 22 Bahman Khaf Hospital and all the people who helped us in conducting this research.

**Journal of BMC Infectious Diseases**

**Dear Editor-in-Chief**

Please, find enclosed our manuscript entitled **“Investigating the trend of demographic changes, mortality, clinical and paraclinical findings of patients hospitalized in the Corona ward, before and after the start of general vaccination of COVID-19 (a retrospective study)”** as a research article for consideration for publication in this journal. We thank our colleagues and all authors for their association and helpful discussions in this study.

We confirm that this manuscript has not been published elsewhere and is not under consideration by another journal. None of the authors have any conflicts of interest to disclose and all authors support submission to this journal.

We, the authors of this article, are all from Iran, and due to the sanctions and financial problems, we request you, honorable professors, to help us with the cost of printing the article.

Sincerely yours,

**Corresponding author: Rasoul Raesi, Email:** [Raesi**.**br881@gmail**.**com](mailto:Raesi.br881@gmail.com)**. ORCID:** 0000-0002-5998-5209
