# Supplementary material for: Investigating the trend of demographic changes, mortality, clinical and paraclinical findings of patients hospitalized in the Corona ward, before and after the start of general vaccination of COVID-19
Source: BMC Infect Dis. 2024 May 13;24:488. doi: 10.1186/s12879-024-09279-z (PMC11089665; doi:10.1186/s12879-024-09279-z)
Supplement: Supplementary file 4 — Supplementary Material 4 [file 12879_2024_9279_MOESM4_ESM.doc]

Investigating the trend of demographic changes, mortality, clinical and paraclinical findings of patients hospitalized in the Corona ward, before and after the start of general vaccination of COVID-19 (a retrospective study)

Running title: Investigation of hospitalized patients in the COVID-19 ward, before and after the general vaccination

Reza Morovatshoar^1^, Kiavash Hushmandi^2^, Sara Orouei^3^, Seyed Hassan Saadat^2^, Rasoul Raesi^4,5*^

1- Molecular Medicine Research Center, Hormozgan Health Institute, Hormozgan University of Medical Sciences, Bandar Abbas, Iran. Email: [Rezamorovatshoar1996@gmail.com](mailto:Rezamorovatshoar1996@gmail.com). ORCID: 0000-0002-0247-7924

2- Nephrology and Urology Research Center, Clinical Sciences Institute, Baqiyatallah University of Medical Sciences, Tehran, Iran. Email: [Kiavash.hushmandi@gmail.com](mailto:Kiavash.hushmandi@gmail.com). Email: [saadat350@gmail.com](mailto:saadat350@gmail.com)

3- Department of psychology, North Tehran branch, Islamic Azad University, Tehran, Iran. Email: [sara_oruei@yahoo.com](mailto:sara_oruei@yahoo.com)

4- Department of Health Services Management, Mashhad University of Medical Sciences, Mashhad, Iran. Email: [Raesi.br881@gmail.com](mailto:Raesi.br881@gmail.com)

5- Department of Nursing, Torbat Jam Faculty of Medical Sciences, Torbat Jam, Iran.

^*^ Corresponding author: Rasoul Raesi, Email: [Raesi.br881@gmail.com](mailto:Raesi.br881@gmail.com). ORCID: 0000-0002-5998-5209

Abstracts

Background: Prioritizing prevention over treatment has been a longstanding principle in the world health system. This study aims to compare the demographic changes, mortality, clinical, and paraclinical findings of patients hospitalized in the Corona ward before and after the start of general vaccination.

Methods: This cross-sectional study utilized the simple random sampling method in 2022, analyzing 300 medical records of patients admitted to the Corona ward at 22 Bahman Khaf Hospital. Data were collected using a checklist with the help of the Medical Care Monitoring System and analyzed using SPSS-22 statistical software and Chi-2 statistical test at a significance level of p<0.05.

Results: Before the start of general vaccination for COVID-19, the majority of patients were hospitalized in the Corona Intensive Care Unit (59.3%), aged between 51-65 years (47.3%), hospitalized for more than 3 days (54%), required intubation (49.3%), had SPO2<93% (60.7%), and exhibited common symptoms such as cough, shortness of breath, and loss of consciousness. Paraclinical findings included positive CRP, decreased lymphocytes, and ground glass opacity (GGO). After the start of general vaccination for COVID-19, most patients were hospitalized in the general care department of Corona (68%), aged between 36-50 years (47.3%), hospitalized for less than three days (66%), required intubation (20%), had SPO2≥93% (77.3%), and exhibited common symptoms such as weakness, headache, and body pain. Paraclinical findings were within the normal range.

Conclusions: General vaccination for COVID-19 has significantly reduced patient mortality and morbidity. Health policymakers should prioritize general vaccination to achieve herd immunity and improve public health.

Keywords: Mortality, morbidity, vaccine, COVID-19, hospital, general vaccination, clinical, findings, Demography.

Background

Prioritizing prevention over treatment has been in the world health system for a long time. The sudden onset of the COVID-19 crisis reaffirmed this priority(1). The consequences and costs caused by this disease had such a profound effect on the economy of countries in the world that it doubled the need for a vaccine as soon as possible. This issue clarified the importance of vaccine production technology as one of the strategic technological capabilities in the current century.(2-4)

Governments have made many efforts to control and suppress this disease, including public education, social distancing policies, screening tests, and vaccine efforts.(1, 5) One of the most important global strategies to control and contain the epidemic of Kovid-19 was the vaccine. Vaccination is the most effective way to control infectious diseases, especially in high-risk groups. In addition to ensuring health and reducing damage and loss of life and ensuring public health, vaccination also leads to a reduction in medical expenses and pharmaceutical costs and as a result economic savings.(6)

During the outbreak of COVID-19, after the clinical trial of a number of vaccines was confirmed, some people were skeptical about getting the vaccine and participating in the nationwide vaccination process. However, after the passage of time and the positive effects of the COVID-19 vaccine on reducing hospitalization and death due to COVID-19, many people showed a positive view and acceptance towards the vaccines approved by the World Health Organization.(7-9)

The results of another study show that injecting two doses of the vaccine significantly reduces the risk of hospitalization. The role of the vaccine in increasing the body's immune system is so impressive that other vaccines are also able to create immunity levels against COVID-19. In this regard, in a study, Wilcox et al investigated the role of the flu vaccine in reducing the hospitalization of people during the outbreak of COVID-19. This study was conducted as a retrospective cohort on 6921 people. Surveys showed that 38% of these people had received the flu vaccine in 2019. The hospitalization rate of people who received the vaccine was very low, to the point that the probability of death of these people was also reduced by 24%.(10)

The results of the studies indicate that the incidence of COVID-19 and the severity of its complications are related to the demographic characteristics of people. So that age is one of the risk factors related to contracting COVID-19. Compared to the young age group (20 to 29 years old), children and the elderly are more likely to be infected with COVID-19. Especially if children are in contact with a patient aged 30 to 39 years or 50 to 59 years, the chance of their infection increases. (9, 11)

The death rate of the disease in February 2020 has been reported from 0.7% to more than 3% depending on the ability of health care.(12) However, in March 2020, the World Health Organization announced the death rate of this disease at 3.4%.(13) Although some studies have mentioned the fatality rate of the disease up to 5%(14). The death rate varies by age. It ranges from 0.2% for ages 10 to 39, to 14.8% for ages over 80. It is 2.8% in men and 1.7% in women. Mortality in healthy people is 0.9%.(15) In Iran, according to a study, the overall mortality rate is 10.5%.(16) The average hospitalization in ICU is 12 days and the mortality rate is 24%.(17)

Nationwide vaccination in Iran started on February 9, 2021 with the priority of medical personnel (18) and after that vaccination was done for high-risk groups such as the elderly and people with underlying diseases. Since the COVID-19 vaccination can be one of the most basic methods of preventing COVID-19 and also reducing mortality. And so far, no study has investigated the effect of COVID-19 vaccination on demographic changes, mortality, clinical and paraclinical findings of patients hospitalized in the corona ward before and after the start of vaccination. This research was carried out as an innovation with the aim of comparing the trend of demographic changes, mortality, clinical and paraclinical findings of patients hospitalized in the corona ward, before and after the start of general vaccination of COVID-19.

Methods

Study design and setting

This descriptive cross-sectional retrospective study was conducted in 2022 using the simple random sampling method by examining the medical records of 300 patients admitted to the Corona Ward at 22 Bahman Hospital in Khaf, located in the northeastern part of Iran.

Study participants and sampling

Since the total number of hospitalized patients diagnosed with coronavirus was known in the investigated period, the sample size was 300 people using Cochran's sample size formula with an error level of 5%. Finally, according to the inclusion and exclusion criteria of the study, by simple random sampling, the 150 medical records of patients with COVID-19 before the start of general vaccination and also the 150 medical records of patients with COVID-19 after the start of general vaccination were examined. 150 medical records were examined in patients with COVID-19 for the period after the start of general vaccination; all had a history of COVID-19 vaccination.

Data collection tool and technique

At this stage, the researcher divided the data collection into two 6-month intervals according to the objectives of the study. The first period is related to before the start of general vaccination of COVID-19 (from May 21, 2020 to January 19, 2021). The second period is related to after the start of general vaccination of COVID-19 (from March 21, 2021 to November 21, 2021). A positive PCR test was considered as the criteria for entering the study, and the incompleteness of the file in terms of the information required for the research was considered as the exclusion criterion. Data collection was done using the Medical Care Monitoring System (MCMC) in the checklist. This checklist consists of three parts, which are: 1- Demographic characteristics (age, gender, Job, education level, marital status, place of residence, underlying diseases, history of smoking, inpatient department, length of hospitalization), 2- Clinical findings (intubation, saturation of peripheral oxygen (SpO2), treatment outcome, fever, cough, dyspnea, weakness, loss of consciousness (LOC), headache, body pain) and 3- paraclinical findings (positive CRP, lymphocytopenia, positive CRP and lymphocytopenia, ground-glass opacity (GGO), patchy consolidation).

Data were analyzed using SPSS version 22 statistical software. To compare the frequency distribution of the variables in the two groups of people under investigation before and after the start of general vaccination of COVID-19, the chi-square test was used. A significance level of p<0.05 was considered.

Results

In this research, 150 patients admitted to the corona ward before the start of general vaccination of COVID-19 and 150 patients admitted to the corona ward after the start of general vaccination of COVID-19 were examined. 150 medical records were examined in patients with COVID-19 for the period after the start of general vaccination; all had a history of COVID-19 vaccination. The comparison of the demographic characteristics of the examined patients before and after the start of general vaccination of COVID-19 showed that there was no significant difference in gender, occupation, education level, marital status and place of residence of the patients before and after the start of general vaccination of COVID-19 (p>0.05). There was a significant difference in the age of patients before and after the start of general vaccination of COVID-19 (p<0.001). So that most of the patients were 51-65 years old before the start of general vaccination of COVID-19, but after the start of general vaccination of COVID-19, most of the people were in the age group of 36-50. Most of the patients (59.3%) were hospitalized in the special ward before the general vaccination of COVID-19 and after the general vaccination of COVID-19 (68%) were hospitalized in the regular ward (p<0.001). Also, before the start of general vaccination of COVID-19, the majority of patients (54%) were hospitalized for more than 3 days, and after the start of general vaccination of COVID-19, most of the patients (66%) were hospitalized for 3 days or less (p<0.001). (Table 1)

Table 1: Comparison of the demographic characteristics of the investigated patients, before and after the start of general vaccination of COVID-19

| Variable | | Before vaccination | After vaccination | Statistical test result |
| --- | --- | --- | --- | --- |
|  |  | Number (percent) | Number (percent) |  |
| Age | 20 years ≥ | 10 (6.7) | 7 (4.7) | 25.24 =x2  0.001 p< |
|  | 21-35 years | 10 (6.7) | 18 (12) |  |
|  | 36-50 years | 27 (18) | 58 (38.7) |  |
|  | 51-65 years | 71 (47.3) | 55 (36.7) |  |
|  | 66-80 years | 32 (21.3) | 12 (8) |  |
| Gender | Male | 93 (62) | 102 (68) | 1.19 =x2  0.28 =p |
|  | Female | 57 (38) | 48 (32) |  |
| Job | Housekeeper | 43 (28.7) | 45 (30) | 7.17=x2  0.13 =p |
|  | Employee | 35 (23.3) | 35 (23.3) |  |
|  | Self-employment | 27 (18) | 41 (27.3) |  |
|  | Retired | 17 (11.3) | 8 (5.3) |  |
|  | Unemployed | 28 (18.7) | 21 (14) |  |
| Education level | Illiterate | 56 (37.3) | 45 (30) | 1.81 =x2  0.41 =p |
|  | Non-academic | 55 (36.7) | 61 (40.7) |  |
|  | Academic | 39 (26) | 44 (29.3) |  |
| Marital status | Single | 51 (34) | 55 (36.7) | 0.32 =x2  0.57=p |
|  | Married | 99 (66) | 95 (63.3) |  |
| Place of residence | City | 89 (59.3) | 102 (68) | 2.44 =x2  0.12 =p |
|  | Village | 61 (40.7) | 48 (32) |  |
| Inpatient department | General | 61 (40.7) | 102 (68) | 22.58 = x2  0.001 p< |
|  | Intensive | 89 (59.3) | 48 (32) |  |
| length of hospitalization | 3 days ≥ | 69 (46) | 99 (66) | 12.18 = x2  0.001 p< |
|  | 3 days < | 81 (54) | 51 (34) |  |

Relative frequency of underlying diseases (diabetes, hypertension, kidney) and history of smoking in the examined patients, before and after the start of general vaccination for COVID-19, did not differ significantly (p>0.05). 74 (49.3%) of the patients had intubation before the start of the general vaccination for COVID-19 and 30 (20%) of them had intubation after the start of the general vaccination for COVID-19 (p<0.001). The Saturation of Peripheral Oxygen (SpO2) level of most of the patients was less than 93 before the general vaccination for COVID-19 (60.7%) and 93 or more after the general vaccination for COVID-19 (77.3%) (p<0.001). The rate of the examined patients was 30.7% before the start of general vaccination for COVID-19, which decreased to 17.3% after the start of general vaccination for COVID-19 (p=0.007). A comparison of clinical findings shows that there was no significant difference in fever in patients before and after COVID-19 general vaccination (p=0.17). However, cough, dyspnea, and loss of consciousness (LOC) before the start of general vaccination for COVID-19 compared to after, and weakness, headache, and body pain after the start of general vaccination for COVID-19 were significantly more than before (p<0.001). (Table 2)

Table 2: Comparison of the relative frequency of underlying diseases, history of smoking, intubation status, Saturation of Peripheral Oxygen (SpO2) level, treatment outcome, and clinical findings in the investigated patients before and after the start of general vaccination against COVID-19.

| Variable | | Before vaccination | After vaccination | Statistical test result |
| --- | --- | --- | --- | --- |
|  |  | Number (percent) | Number (percent) |  |
| Diabetes disease | No | 65 (43.3) | 69 (46) | 0.22 = x2  0.64 =p |
|  | Yes | 85 (56.7) | 81 (54) |  |
| Hypertension disease | No | 76 (50.7) | 64 (42.7) | 1.93 = x2  0.17=p |
|  | Yes | 74 (49.3) | 86 (57.3) |  |
| kidney disease | No | 147 (98) | 149 (99.3) | 1.01 = x2  0.31 =p |
|  | Yes | 3 (2) | 1 (0.7) |  |
| Smoking history | No | 81 (54) | 93 (62) | 1.97 = x2  0.16 =p |
|  | Yes | 69 (46) | 57 (38) |  |
| Intubation | No | 76 (50.7) | 120 (80) | 28.49= x2  0.001 p< |
|  | Yes | 74 (49.3) | 30 (20) |  |
| Saturation of Peripheral Oxygen (SpO2) | 93> | 91 (60.7) | 34 (22.7) | 44.56 = x2  0.001 p< |
|  | 93≤ | 59 (39.3) | 116 (77.3) |  |
| Treatment outcome | Improvement and discharge | 104 (69.3) | 124 (82.7) | 7.31= x2  0.007=p |
|  | Death | 46 (30.7) | 26 (17.3) |  |
| Fever | No | 11 (7.3) | 18 (12) | 1.87 = x2  0.17 =p |
|  | Yes | 139 (92.7) | 132 (88) |  |
| Cough | No | 0 (0) | 54 (36) | 65.85 = x2  0.001 p< |
|  | Yes | 150 (100) | 96 (64) |  |
| Dyspnea | No | 7 (4.7) | 123 (82) | 182.66 = x2  0.001 p< |
|  | Yes | 143 (95.3) | 27 (18) |  |
| Weakness | No | 149 (99.3) | 127 (84.7) | 21.92 = x2  0.001 p< |
|  | Yes | 1 (0.7) | 23 (15.3) |  |
| Loss of consciousness (LOC) | No | 125 (83.3) | 140 (93.3) | 7.28 = x2  0.007=p |
|  | Yes | 25 (16.7) | 10 (6.7) |  |
| Headache | No | 139 (92.7) | 117 (78) | 12.89 = x2  0.001 p< |
|  | Yes | 11 (7.3) | 33 (22) |  |
| Body pain | No | 150 (100) | 120 (80) | 33.33 = x2  0.001 p< |
|  | Yes | 0 (0) | 30 (20) |  |

The comparison of laboratory and radiological findings in Table (3) shows that the laboratory findings of most patients before the start of general vaccination were positive CRP and lymphocytopenia and normal after the start of vaccination (p<0.001). The findings of radiology were also normal in most patients before the start of general vaccination of COVID-19, Ground-glass opacity (GGO), and after the start of general vaccination (p<0.001).

Table 3: Comparison of laboratory and radiological findings in the examined patients, before and after the start of general vaccination of COVID-19

| Variable | | Before vaccination | After vaccination | Statistical test result |
| --- | --- | --- | --- | --- |
|  |  | Number (percent) | Number (percent) |  |
| Laboratory findings | Normal | 49 (32.7) | 49 (32.7) | 39.25 = x2  0.001 p< |
|  | positive CRP | 49 (32.7) | 49 (32.7) |  |
|  | Lymphocytopenia | 49 (32.7) | 49 (32.7) |  |
|  | positive CRP and Lymphocytopenia | 49 (32.7) | 49 (32.7) |  |
| Radiological findings | Normal | 49 (32.7) | 49 (32.7) | 62.59= x2  0.001 p< |
|  | Ground-glass opacity (GGO) | 49 (32.7) | 49 (32.7) |  |
|  | Patchy consolidation | 49 (32.7) | 49 (32.7) |  |

Discussion

The present study was conducted with the aim of comparing the trend of demographic changes, mortality, clinical and paraclinical findings of patients admitted to the corona ward, before and after the start of general vaccination of COVID-19. The most important findings of the present study indicate that before the start of general vaccination for COVID-19, the majority of patients were hospitalized in the Corona Intensive Care Unit (59.3%), aged between 51-65 years (47.3%), hospitalized for more than 3 days (54%), required intubation (49.3%), had SPO2<93% (60.7%), and exhibited common symptoms such as cough, shortness of breath, and loss of consciousness. Paraclinical findings included positive CRP, decreased lymphocytes, and ground glass opacity (GGO). After the start of general vaccination for COVID-19, most patients were hospitalized in the general care department of Corona (68%), aged between 36-50 years (47.3%), hospitalized for less than three days (66%), required intubation (20%), had SPO2≥93% (77.3%), and exhibited common symptoms such as weakness, headache, and body pain. Paraclinical findings were within the normal range.

The findings of the present study showed that before the general vaccination of COVID-19, most of the patients hospitalized in corona wards were in the age range of 51-65 years. However, after the start of general vaccination against COVID-19, most of the patients hospitalized in corona wards were in the age range of 36-50 years.

In line with this finding of the present study, the results of the studies A. Christie et al (2021) (19), M. L. Salomão et al. (2022) (20) as well as Emre Özgen et al. (2023) (21) showed that general vaccination of COVID-19 was associated with a change in the age range of patients hospitalized in corona wards. So that after the start of general vaccination of COVID-19, most of the patients hospitalized in corona wards were in the age group of less than 50 years.

But the results of the study K. Dooling et al. (2021) (22) as well as the results of the study by H. Rossman et al. (2021) (23) are not in line with this finding of the present study. So that the results of their studies showed that the general vaccination of COVID-19 is not related to the change in the age range of hospitalized patients. Also, most of the patients admitted to the Corona wards were elderly people over 60 years old. This discrepancy in research findings can be related to the different geographical environment in the studies as well as the type and number of hospitalized patients.

The findings of the present study showed that most of the patients were hospitalized in the Corona Intensive Care Department before the general vaccination of COVID-19. After the start of the general vaccination of COVID-19, most of the patients were hospitalized in the normal care department of Corona. The results of the study of M. Moffa et al. (2022) (24) as well as the results of the study M. Fogolari et al. (2022) (25) is in line with this finding of the present study. The results of their studies showed that after the start of general vaccination of COVID-19, most of the patients were hospitalized in the general care departments for corona patients. So that the number of patients hospitalized in the special corona wards has decreased.

But the results of the study of M. Özsoy et al. (2023) (26) as well as the results of the study of B. Ngo et al. (2021) (27) is not in line with this finding of the present study. So that the results of their study showed that there is no connection between the start of general vaccination of COVID-19 and the type of inpatient department of corona patients. This discrepancy in research findings can be related to the different geographical environment in the studies as well as the type and number of hospitalized patients.

The findings of the present study showed that before the general vaccination of COVID-19, most of the patients were hospitalized for more than 3 days in the care units for corona patients. Also, the findings showed that after the start of general vaccination for COVID-19, most of the patients were hospitalized in the care units for corona patients for less than three days.

In line with this finding of the current research, the results of the study of M. Tenforde et al. (2021) (28) and the results of the study by Aakashneel Bhattacharya et al. (2021) (29) showed that the start of general vaccination of COVID-19 is related to the number of days of hospitalization of patients in corona wards. so that after the start of the general vaccination of COVID-19, the number of days of hospitalization of patients in corona wards has decreased.

However, the results of the study of G. Suleyman et al. (2022) (30) and the results of the study of Anshuman Srivastava et al. (2022) (31) are not in line with this finding of the present study. So that the results of their study showed that there is no relationship between the start of general vaccination for COVID-19 and the number of days of hospitalization of patients in the care departments for corona patients.

The findings of the present study showed that the number of patients requiring intubation had decreased after the start of general vaccination against COVID-19. In line with this finding of the present study, the results of the study by C. Bezzio et al. (2020) (32) as well as the results of the study by Cristiane de Freitas Paganoti et al. (2022) (33) showed that the start of general vaccination of COVID-19 has been associated with a decrease in the need for intubation and also a decrease in the need for hospitalization in special corona wards.

The findings of the present study showed that the percentage of oxygen saturation (SPO2) in most patients was less than 93% before the start of the general vaccination of COVID-19 and more than 93% after the start of the general vaccination of COVID-19. In line with this finding of the present study, the results of Linzy Houchen-Wolloff et al. (2021) (34) as well as the results of the study of Ulfa Husnul Fata et al. (2022) (35) showed that the start of general vaccination of COVID-19 was associated with an increase in the percentage of oxygen saturation in patients hospitalized in corona wards.

The findings of the present study showed that the mortality rate in patients hospitalized in the care units for Corona patients decreased after the start of general vaccination for COVID-19. The results of the study by R. Kempker et al. (2022) (36) and the results of the study by P. Moreno-Nunez et al. (2022) (37) are in line with this finding of the present study. The results of their study showed that the start of general vaccination for COVID-19 was associated with a decrease in mortality in patients hospitalized in COVID-19 wards.

The findings of the present study showed that before the general vaccination of COVID-19, cough, dyspnea, and loss of consciousness were among the common symptoms of patients hospitalized in corona wards. However, after the start of the general vaccination of COVID-19, general weakness, headache, and body pain were among the common symptoms of patients hospitalized in corona wards. The results of the study of Zunaira Khan et al. (2022) (38) are in line with this finding of the present study, as the results of their study showed that: after the start of the general vaccination of COVID-19, general weakness, headache, and acute myelopathy were among the common symptoms of patients hospitalized in corona wards.

Also, the results of the study by L. Bonifácio et al. (2022) (39) are in line with this finding of the present study. The results of their study showed that: before the general vaccination of COVID-19, common symptoms like cough, dyspnea, and loss of consciousness were common, but after the vaccination, general weakness, headache, and muscle weakness emerged.

The findings of the present study showed that the laboratory results of most of the patients hospitalized in the care units for corona patients before the start of general vaccination of COVID-19 included positive CRP and a decrease in lymphocytes. However, after the start of the general vaccination of COVID-19, the laboratory findings of most of the patients hospitalized in the care units for corona patients were normal.

The results of the study of H. Fu et al. (2020) (40) are in line with this finding of the present study, in such a way that the results of their study showed that: after the start of general vaccination, CRP levels decreased significantly and lymphocyte counts increased in COVID-19 patients. Also, the results of the study of H. Akbari et al. (2020) (41) are in line with this finding of the present study. The results of their study showed that: before the start of general vaccination, most COVID-19 patients had a decrease in lymphocytes and an increase in CRP, but after the start of vaccination, these results reversed.

The findings of the present study showed that the radiology results of most of the patients hospitalized in the care departments of Corona patients before the start of general vaccination of COVID-19 were ground glass opacity (GGO). However, after the start of general vaccination against COVID-19, the radiology results of most of the patients hospitalized in the care units for corona patients were normal.

The results of the study of Mamatha Reddy D. Cozzi et al. (2021) (42) are in line with this finding of the present study in such a way that the results of their study showed that: before the start of general vaccination against COVID-19, most hospitalized patients had ground-glass opacities (GGO) on CT, but after the vaccination, GGO results decreased. Also, the results of the study of Jufriadif Na'am et al. (43) (2021) are in line with this finding of the present study in such a way that the results of their study showed that: before the start of general vaccination against COVID-19, most hospitalized patients had radiology results showing ground glass opacity (GGO) in their thorax.

Limitations and recommendation

Among the limitations of the study were the short study period and the small number of samples under investigation. It is suggested to conduct future studies over a longer period of time and with a larger sample size. Another limitation of the current study was the newness of the MCMC system, which was associated with limitations such as not recording a number of variables. Therefore, access to all demographic, clinical, and paraclinical variables of the patients was not possible.

Conclusion

The research findings reveal significant shifts in the clinical profile of COVID-19 patients before and after the general vaccination for COVID-19. These findings have several implications for health policymakers. First, they underscore the importance of prioritizing COVID-19 vaccination, particularly for high-risk groups such as the elderly and those with comorbidities. Second, the shift in patient demographics and clinical presentation highlights the need for ongoing public awareness campaigns to promote vaccination uptake and address concerns about vaccine safety and efficacy.

Third, the research emphasizes the critical role of healthcare providers in initiating discussions about COVID-19 vaccines with patients and acting as role models by getting vaccinated themselves.

In light of these findings, health policymakers should continue to prioritize and promote COVID-19 vaccination efforts, provide training and guidelines for healthcare providers, and monitor vaccine effectiveness and safety. Additionally, efforts should be made to address disparities in vaccination uptake and ensure that accurate information about COVID-19 vaccines is accessible to all segments of the population. By implementing these recommendations, policymakers can contribute to the ongoing efforts to mitigate the impact of the COVID-19 pandemic and protect public health.

Declarations

Ethics approval and consent to participate

All procedures performed in the study involving human participants were by the ethical standards of the institutional and national research committee and with the 1975 Helsinki Declaration and its later amendments or comparable ethical standards. This study was approved by the Biomedical Research Ethics Committee of Mashhad University of Medical Sciences, which issued the study’s code of ethics (IR.MUMS.REC.1401.050.).

Consent for publication

In this study, no intervention was performed on any of the patients. Also, there were no tissue samples in this research. Rather, only the information needed in this research was extracted from the medical files of the patients in the medical records unit of the hospital. Therefore, this study did not require a statement confirming that informed consent was obtained from all individuals and/or their legal guardian(s).

To comply with ethical considerations in this research, the patient's medical record information was kept confidential and other people could not access this information. The names and surnames of the participants were not used for data collection, and data collection was done after obtaining the code of ethics from Mashhad University of Medical Sciences.

Availability of data and materials

The data that support the findings of this study are available from the corresponding author upon reasonable request.

Competing interests

The authors declare no conflict of interest, financial or otherwise.

Funding

This research was done with the financial support of Mashhad University of Medical Sciences.

Authors' contributions

"RM and KH analyzed and interpreted the data. RR, SO, SHS contributor in writing the manuscript. All authors read and approved the final manuscript."

Acknowledgements

We would like to express our sincere thanks and appreciation to the honorable research assistant of Mashhad University of Medical Sciences, the honorable officials of 22 Bahman Khaf Hospital and all the people who helped us in conducting this research.

References

1. Hushmandi K, Tabatabaee SS, Saghari S, Zandieh MA, Raesi R. Investigating the reasons for the unwillingness to get vaccinated against COVID-19 in the General Population. The Open Public Health Journal. 2023;16(1).

2. Sumner A, Hoy C, Ortiz-Juarez E. Estimates of the Impact of COVID-19 on Global Poverty: United Nations University World Institute for Development Economics Research; 2020.

3. Narula R. Policy opportunities and challenges from the COVID-19 pandemic for economies with large informal sectors. Journal of International Business Policy. 2020;3(3):302-10.

4. Li J, Pega F, Ujita Y, Brisson C, Clays E, Descatha A, et al. The effect of exposure to long working hours on ischaemic heart disease: A systematic review and meta-analysis from the WHO/ILO Joint Estimates of the Work-related Burden of Disease and Injury. Environment international. 2020;142:105739.

5. Saebniya S, Karimi F. The effect of corona heart disease (Covid-19) on personal and work performance (Case study of small and medium businesses in Ardabill province). Journal of Accounting and Management Perspective. 2020;4:83-93.

6. MOKHBER ALSAFA L, Forghani S, HAGHIGHI S, AFGHAM NIA MR. SATISFACTION AND ATTITUDE OF THE INDUSTRIAL CATTLE RANCHERS TOWARDS BRUCELLOSIS , ANTHRAX AND FMD VACCINES OF RAZI INSTITUTE 2004-2005. VETERINARY RESEARCHES BIOLOGICAL PRODUCTS (PAJOUHESH-VA-SAZANDEGI). 2017;30(2 (115)):-.

7. Bono SA, Faria de Moura Villela E, Siau CS, Chen WS, Pengpid S, Hasan MT, et al. Factors affecting COVID-19 vaccine acceptance: an international survey among low-and middle-income countries. Vaccines. 2021;9(5):515.

8. Abbasi Z, Moghadaci A, Mohammadnahal L, Sangrizeh FH, Gholami MH, Baeelashaki R, et al. Investigating how Interleukin 6 Serum Level, Blood Group Type, and Underlying Diseases are Associated in Patients Admitted to the COVID-19 Intensive Care Unit: A Retrospective Study. The Open Public Health Journal. 2023;16(1).

9. Raesi R, Abbasi Z, Raei M, Hushmandi K. The relationship between the incidence of COVID-19 with the underlying diseases in hospitalized patients. EBNESINA. 2022;24(3):75-80.

10. Wilcox CR, Islam N, Dambha-Miller H. Association between influenza vaccination and hospitalisation or all-cause mortality in people with COVID-19: a retrospective cohort study. BMJ open respiratory research. 2021;8(1):e000857.

11. Liu T, Liang W, Zhong H, He J, Chen Z, He G, et al. Risk factors associated with COVID-19 infection: a retrospective cohort study based on contacts tracing. Emerging microbes & infections. 2020;9(1):1546-53.

12. Ji Y, Ma Z, Peppelenbosch MP, Pan Q. Potential association between COVID-19 mortality and health-care resource availability. The Lancet Global Health. 2020;8(4):e480.

13. Organization WH. WHO Director-General’s opening remarks at the media briefing on COVID-19-11 March 2020. Geneva, Switzerland; 2020.

14. Li Lq, Huang T, Wang Yq, Wang Zp, Liang Y, Huang Tb, et al. COVID‐19 patients' clinical characteristics, discharge rate, and fatality rate of meta‐analysis. Journal of medical virology. 2020;92(6):577-83.

15. Cirrincione L, Plescia F, Ledda C, Rapisarda V, Martorana D, Moldovan RE, et al. COVID-19 pandemic: Prevention and protection measures to be adopted at the workplace. Sustainability. 2020;12(9):3603.

16. Zali A, Gholamzadeh S, Mohammadi G, Looha MA, Akrami F, Zarean E, et al. Baseline characteristics and associated factors of mortality in COVID-19 Patients; an analysis of 16000 cases in Tehran, Iran. Archives of academic emergency medicine. 2020;8(1).

17. Mehrtash B, Siahoosh MB. A Review on Epidemiology, Pathophysiology and Clinical Manifestations of COVID-19 Infection in Order to Guide Policy Making and Promotion of Knowledge, Attitude and Practice of the Society Associated with COVID-19: A Validity Review. Journal of Rafsanjan University of Medical Sciences. 2021;19(11):1195-224.

18. Bagheri Sheykhangafshe F. Coronavirus 2019 (COVID-19) vaccination: Prioritizing people with psychological disorders. Health Monitor Journal of the Iranian Institute for Health Sciences Research. 2021;20(2):243-5.

19. Christie A, Henley SJ, Mattocks L, Fernando R, Lansky A, Ahmad FB, et al. Decreases in COVID-19 cases, emergency department visits, hospital admissions, and deaths among older adults following the introduction of COVID-19 vaccine—United States, September 6, 2020–May 1, 2021. Morbidity and Mortality Weekly Report. 2021;70(23):858.

20. Salomão MLM, Machado MN, Fernandes EG, Queiroz F, Mendes LdM, Tuckumantel MdS, et al. Hospitalizations for COVID-19 in a tertiary hospital in Brazil: a parallel with vaccination. Revista do Instituto de Medicina Tropical de São Paulo. 2022;64.

21. ÖZGEN E, YAZICIOĞLU B, BİLGİN M, ORUÇ MA. Effect of Covid-19 Vaccination On Hospitalizations. Black Sea Journal of Health Science. 2023;6(2):246-52.

22. Dooling K, Marin M, Wallace M, McClung N, Chamberland M, Lee GM, et al. The Advisory Committee on Immunization Practices’ updated interim recommendation for allocation of COVID-19 vaccine—United States, December 2020. Morbidity and Mortality Weekly Report. 2021;69(51-52):1657.

23. Rossman H, Shilo S, Meir T, Gorfine M, Shalit U, Segal E. COVID-19 dynamics after a national immunization program in Israel. Nature medicine. 2021;27(6):1055-61.

24. Moffa MA, Shively NR, Walsh TL, editors. Characteristics of postvaccination coronavirus disease 2019 hospitalizations prior to booster vaccines. Open forum infectious diseases; 2022: Oxford University Press US.

25. Fogolari M, Francesconi M, De Florio L, Giovanetti M, Veralli R, De Flora C, et al. SARS-CoV-2 Variants in COVID-19 Disease: A Focus on Disease Severity and Vaccine Immunity in Patients Admitted to the Emergency Department. Journal of Personalized Medicine. 2022;12(12):2001.

26. Özsoy M, Cesur S, HATİPOGLU CA, ERDİNÇ Ş, ERTEM GT, KINIKLI S. Investigating clinical and laboratory findings and mortality rates among vaccinated and unvaccinated COVID-19 inpatients. Anatolian Current Medical Journal. 2023;5(1):29-33.

27. Ngo BT, Marik P, Kory P, Shapiro L, Thomadsen R, Iglesias J, et al. The time to offer treatments for COVID-19. Expert opinion on investigational drugs. 2021;30(5):505-18.

28. Tenforde MW, Self WH, Naioti EA, Ginde AA, Douin DJ, Olson SM, et al. Sustained effectiveness of Pfizer-BioNTech and Moderna vaccines against COVID-19 associated hospitalizations among adults—United States, March–July 2021. Morbidity and Mortality Weekly Report. 2021;70(34):1156.

29. Bhattacharya A, Ranjan P, Ghosh T, Agarwal H, Seth S, Maher GT, et al. Evaluation of the dose-effect association between the number of doses and duration since the last dose of COVID-19 vaccine, and its efficacy in preventing the disease and reducing disease severity: A single centre, cross-sectional analytical study from India. Diabetes & Metabolic Syndrome: Clinical Research & Reviews. 2021;15(5):102238.

30. Suleyman G, Fadel R, Alsaadi A, Sueng LN, Ghandour A, Alkhatib A, et al., editors. Progression to critical illness and death in patients with breakthrough hospitalizations. Open Forum Infectious Diseases; 2022: Oxford University Press.

31. SRIVASTAVA A, SHARMA A, JHAMB R, GIRI S, AGGARWAL N. Vaccination Status and Outcome of Patients at a Dedicated COVID-19 Centre, Delhi, India: A Retrospective Study. Journal of Clinical & Diagnostic Research. 2022;16(12).

32. Bezzio C, Saibeni S, Variola A, Allocca M, Massari A, Gerardi V, et al. Outcomes of COVID-19 in 79 patients with IBD in Italy: an IG-IBD study. Gut. 2020;69(7):1213-7.

33. de Freitas Paganoti C, Alkmin da Costa R, Papageorghiou AT, da Silva Costa F, Quintana SM, Graziela de Godoi L, et al. COVID-19 vaccines confer protection in hospitalized pregnant and postpartum women with severe COVID-19: a retrospective cohort study. Vaccines. 2022;10(5):749.

34. Houchen-Wolloff L, Daynes E, Gerlis C, Chaplin E, Gardiner N, Singh S. A Simple Sit-to-Stand Test to Detect Exercise-Induced Oxygen Desaturation in Patients Being Discharged from Hospital with COVID-19. TP101 TP101 REHABILITATION IN THE TIME OF COVID-19: American Thoracic Society; 2021. p. A4122-A.

35. Fata UH, Febriana L. Oxygen Saturation (SPO2) in Covid-19 Patients. Jurnal Ners dan Kebidanan (Journal of Ners and Midwifery). 2021;8(3):290-4.

36. Kempker RR, Rebolledo PA, Rollin F, Gurbani S, Schechter MC, Wilhoite D, et al. High Mortality among Older Patients Hospitalized with COVID-19 during the First Pandemic Wave. medRxiv. 2022:2022.06. 16.22276514.

37. Moreno-Nunez P, Bueno-Cavanillas A, San Jose-Saras D, Vicente-Guijarro J, Fernández Chávez AC, Aranaz-Andrés JM, et al. How Does Vaccination against SARS-CoV-2 Affect Hospitalized Patients with COVID-19? Journal of Clinical Medicine. 2022;11(13):3905.

38. Khan Z, Khattak AA, Rafiq N, Amin A, Abdullah M. Interstitial lung disease and transverse myelitis: a possible complication of COVID-19 vaccine. Cureus. 2022;14(2).

39. Bonifácio LP, Csizmar VN, Barbosa-Júnior F, Pereira AP, Koenigkam-Santos M, Wada DT, et al. Long-term symptoms among COVID-19 survivors in prospective cohort study, Brazil. Emerging Infectious Diseases. 2022;28(3):730.

40. Fu H, Xu H, Zhang N, Xu H, Li Z, Chen H, et al. Association between Clinical, Laboratory and CT Characteristics and RT-PCR Results in the Follow-up of COVID-19 patients. MedRxiv. 2020:2020.03. 19.20038315.

41. Akbari H, Tabrizi R, Lankarani KB, Aria H, Vakili S, Asadian F, et al. The role of cytokine profile and lymphocyte subsets in the severity of coronavirus disease 2019 (COVID-19): a systematic review and meta-analysis. Life sciences. 2020;258:118167.

42. Cozzi D, Cavigli E, Moroni C, Smorchkova O, Zantonelli G, Pradella S, et al. Ground-glass opacity (GGO): a review of the differential diagnosis in the era of COVID-19. Japanese journal of radiology. 2021;39(8):721-32.

43. Na'am J, Pranata FS, Hidayat R, Adif AM, Ellyzarti E. Automated Identification Model of Ground-Glass Opacity in CT-Scan Image by COVID-19. International Journal on Advanced Science, Engineering and Information Technology. 2021;11(2):595-602.
