# Supplementary material for: Investigating the trend of demographic changes, mortality, clinical and paraclinical findings of patients hospitalized in the Corona ward, before and after the start of general vaccination of COVID-19
Source: BMC Infect Dis. 2024 May 13;24:488. doi: 10.1186/s12879-024-09279-z (PMC11089665; doi:10.1186/s12879-024-09279-z)
Supplement: Supplementary file 3 — Supplementary Material 3 [file 12879_2024_9279_MOESM3_ESM.docx]

REVIEWER REPORTS

Reviewer Comments:

Reviewer 1
In this retrospective study, authors report their findings about demographic changes, mortality, clinical and paraclinical findings of patients hospitalized in the Corona ward, before and after the start of general vaccination of COVID-19 .

Greetings and respect to my dear and honorable teacher. Thank you very much for your kindness, love and support. Thank you very much for your efforts. Your valuable comments led to the quantitative and qualitative improvement of our article. We have done everything you said and highlighted it in yellow. Yours sincerely

The vaccination status of the second group of people has not been determined.

Thank you for your valuable guidance, dear professor. It was added and highlighted in yellow color. 150 medical records were examined in patients with COVID-19 for the period after the start of general vaccination; all had a history of COVID-19 vaccination. Yours sincerely

The day of onset of the symptoms of both groups should match according to the effect on the severity of symptoms and hypoxia,

Thank you for your valuable guidance, dear professor. Again, like your previous comment, I must say that you are exactly right. But again, because the MCMC system was a new system and had just been launched, it had many limitations. Also, the day of onset of the patients' symptoms was not available in this system so that we could use it as an investigated variable. Therefore, the day of onset of symptoms in both groups was also unclear and it was not possible to collect data for this variable. According to your honorable opinion, I add this item as one of the limitations of the study at the end of the article to solve the problem.

The number of tables is large, they should be merged as much as possible.

Thank you for your valuable guidance, dear professor. Tables were merged together and the number of tables was reduced from 5 to 3.

Reviewer 2
Dear Author(s);
Your study is a valuable study showing the value of vaccination against COVID 19. I think it is necessary to publish your article in these times when the voices of opponents of vaccination are loud. I congratulate author(s). I made a few suggestions to make the article more understandable and ensure its fluency.

Greetings and respect to my dear and honorable teacher. Thank you very much for your kindness, love and support. Thank you very much for your efforts. Your valuable comments led to the quantitative and qualitative improvement of our article. We have done everything you said and highlighted it in yellow. Yours sincerely

1. In the study, the medical records of 150 COVID-19 patients before vaccination and 150 COVID-19 patients after vaccination were examined. How were these patients identified? It should be explained. Was a random systematic sampling method used? (If not used, this may be a cause of bias.) For example, stratified or mixed sampling or other.... While reading the article, for example: before / after vaccination makes one think of a 'stratified sample', it is not known on what basis the selection of 150 patients was based. etc

Thank you for your valuable advice, dear professor. It was modified and highlighted in yellow color in the methodology section.

2. In the 'Data collection tool and technique' section 'This checklist included demographic characteristics, clinical and paraclinical findings.' It would be appropriate to provide details.

Thank you for your valuable guidance, dear professor. It was added and highlighted in yellow color.

3. Spelling errors such as 17/0 =p and 007/0 =p should be checked in the tables.

Thank you for your valuable advice, dear professor. It was modified and highlighted.
4. Having 'total' in the tables will make it easier to read. I leave this suggestion to the authors' discretion.
However
*'Marriage status' is given as 149 people.
*The total number of patients with hypertension (for the post-vaccination group) does not 150.
*'Inpatient department' status is incompatible with the total number of patients (150).
should be checked and corrected.

Thank you for your valuable advice, dear professor. It was modified and highlighted in yellow color

5. The first paragraph of the discussion section should describe the results of your study.
in the first paragraph
*Study purpose (specified).
In continuation
*Data with statistical significance should be written.
*Data that does not have statistical significance but needs to be emphasized can also be written.
    Add the first paragraph to the discussion section...

Thank you for your valuable guidance, dear professor. The first paragraph of the discussion section was written and modified according to the pattern you said.
I wish you success in your work...
Thank you very much for your kindness, love and support

Reviewer 3
Dear author(s), Thank you for your hard work.
I think it would be appropriate to print the work after correcting the spelling errors.

Greetings and respect to my dear and honorable teacher. Thank you very much for your kindness, love and support. Thank you very much for your efforts. Your valuable comments led to the quantitative and qualitative improvement of our article. Editing and writing errors in the entire article were checked and corrected.
